# Effects of combined traditional Chinese medicine therapy in patients of lower limbs injuries with osteoporosis: A retrospective paired cohort study

**DOI:** 10.1097/MD.0000000000036489

**Published:** 2023-12-08

**Authors:** Yu-Hua Lu, Chi-Hsiang Chung, Chien-Jung Lin, Li-Jen Tsai, Kuang-Chung Shih, Chieh-Hua Lu, Wu-Chien Chien

**Affiliations:** a Department of Athletic, National Taiwan University, Taipei, Taiwan, ROC; b School of Public Health, National Defense Medical Center, Taipei, Taiwan, ROC; c Taiwanese Injury Prevention and Safety Promotion Association, Taipei, Taiwan, ROC; d Department of Chinese Medicine, Tri-Service General Hospital, National Defense Medical Center, Taipei, Taiwan, ROC; e Graduate Institute of Life Sciences, National Defense Medical Center, Taipei, Taiwan, ROC; f Division of Endocrinology and Metabolism, Department of Medicine, Cheng-Hsin General Hospital, Taipei, Taiwan, ROC; g Division of Endocrinology and Metabolism, Department of Internal Medicine, Tri-Service General Hospital, School of Medicine, National Defense Medical Center, Taipei, Taiwan, ROC; h Department of Medical Research, Tri-Service General Hospital, National Defense Medical Center, Taipei, Taiwan, ROC.

**Keywords:** lower limb injuries, osteoporosis, Taiwan National Health Insurance Database, traditional Chinese medicine

## Abstract

Studies have confirmed that the health hazards of patients with lower limb injuries combined with osteoporosis are more obvious. This study is mainly based on the Taiwan National Health Insurance Database, and through big data analysis, it shows that the combined treatment of traditional Chinese medicine (TCM) is helpful to the health of patients with lower limb injuries combined with osteoporosis. A total of 9989 combined TCM-treated patients and 19,978 2:1 sex-, age-, and index-year-matched controls who did not receive TCM treatment were selected from the Taiwan National Health Insurance Database. Cox proportional hazards analyzes were performed to compare fracture surgery, inpatient, and all-cause mortality during a mean follow-up period of 17 years. A total of 5406/8601/2564 enrolled-subjects (14.11%/25.46%/5.53%) had fracture surgery/inpatient/all-cause mortality, including 1409/2543/552 in the combined TCM group (14.11%/25.46%/5.53%) and 3997/6058/2012 in the control group (20.01%/30.32%/10.07%). Cox proportional hazard regression analysis showed a lower rate of fracture surgery, inpatient and all-cause mortality for subjects in the combined TCM group (adjusted hazard ratios [HR] = 0.723; 95% confidence intervals [CI] = 0.604–0.810, *P* < .001; adjusted hazard ratios [HR] = 0.803; 95% CI = 0.712–0.950, *P* = .001; adjusted HR = 0.842; 95% CI = 0.731–0.953, *P* = .007, respectively). After 10 years of follow-up, the cumulative incidence of fracture surgery in patients combining TCM treatment seems to be half of that without combining TCM treatment those are shown in Kaplan–Meier analysis with statistically significant (log rank, *P* < .001, *P* < .001, and *P* = .010, respectively). This study hopes to provide clinicians with the option of combined TCM treatment for patients of lower limbs injuries combined with osteoporosis, so that such patients will be associate with a lower risk of fracture surgery, inpatient or all-cause mortality.

## 1. Introduction

The World Health Organization has determined that osteoporosis is the second most important epidemic in the world, second only to cardiovascular diseases.^[1]^ A fracture caused by osteoporosis will occur almost every 3 seconds.^[2]^ It is estimated that by 2050, the Asian region will suffer from osteoporosis will cause hip fractures in as many as 3.25 million people.^[3]^

There are many reasons for osteoporosis. Excluding related underlying disease, old age, lack of activity or sun exposure are common risk factors for osteoporosis.^[4]^ Once the lower limbs are injured, the clinical recommendation is to let the patient rest and stay in bed, which will lead to rapid muscle strength atrophy.^[5]^ Generally, doctors will recommend starting muscle strength training as soon as possible.^[6]^ Strengthening the patient’s lower limb muscle strength can prevent or reduce the occurrence of falls in patients with high risk tendencies which resulted in bony fracture, disability or death.^[7]^

At present, many studies have explored that osteoporosis is likely to cause fractures and lead to disability in patients.^[8]^ Combining the treatment of traditional Chinese medicine (TCM) can improve osteoporosis and the harm caused by osteoporosis to health.^[9]^ The health hazards are more obvious when osteoporosis patients are combined with lower limbs injuries.^[2]^ However, there are relatively few studies discussing whether the combination of TCM treatment can reduce the risk of fracture surgery, inpatient or death for patients of lower limbs injuries combined with osteoporosis. Therefore, this study is mainly based on the Taiwan National Health Insurance Database (TNHID), and through the analysis of big data, it has been confirmed that combined TCM treatment is helpful for patients of lower limb injuries combined with osteoporosis.

## 2. Materials and methods

### 2.1. Data source

Patients with lower limbs injuries and osteoporosis were collected from TNHID. We used data from TNHID to investigate whether combined TCM could reduce fracture surgery, inpatient, or death in patients with lower limbs injuries and osteoporosis over a 17-year period (2000–2017). In 1995, Taiwan launched the National Health Insurance Plan. As of 2009, it has contracted with 97% of medical providers in Taiwan, which has a population of about 23 million and 99% of the total population of Taiwan. TNHID uses the International Classification of Diseases, 9th Revision, Clinical Modification (ICD-9-CM) to record classifiable diagnoses.^[10]^ All diagnoses of lower limbs injuries and osteoporosis are made by specialists. The NHI randomly reviewed every 100 outpatient visits and every 20 hospitalization-related records to verify diagnostic accuracy. Numerous studies have confirmed the diagnostic accuracy and validity of TNHID.^[11–13]^

### 2.2. Study design and sample participants

Our study used a retrospective paired cohort design. From January 1, 2000 to December 31, 2015, according to the codes ICD-9-CM 733.XX (osteoporosis) and ICD-9-CM 820–824, 835, 836, 843, 844, 890, 891, 897, 904, 916, 928, 956, 959 (lower limbs injuries) respectively, select the diagnosis of osteoporosis and lower limbs injuries. According to these ICD-9-CM codes, each enrolled patient had at least 3 outpatient visits during the study period, and patients who received <3 TCM treatments and were younger than 18 years old were excluded and patients with known bone metabolic diseases and long-term steroid used (more than 60 days in 18 months) were excluded. All lower extremity injuries and osteoporosis diagnoses were made by professionally certified medical professionals and herbalists, also including anatomical therapeutic chemical—code and average follow-up time and other information were in Table S1, Supplemental Digital Content, http://links.lww.com/MD/L8. Catastrophic illness is an acute or long-term illness that threatens life or threatens severe residual disability, such as heart attack, stroke, or cancer. Covariates included Charlson comorbidity index minus osteoporosis, lower limbs injuries, level of care, sex, and age.^[12]^

### 2.3. Outcome measures

According to the NHI program, we mainly track when patients have lower limb fracture surgery, inpatient or death due to any reasons. The tracking period until the end of 2017 is outcome measures to see if combined with TCM treatment can reduce the incidence of these events.

### 2.4. Statistical analysis

All statistical analyzes were performed using SPSS software version 22 for Windows (SPSS Inc., Chicago, IL). Chi-square and *t*-tests were used to assess the distribution of categorical and continuous variables, respectively. Multivariate Cox proportional hazards regression analysis was used to determine the risk of fracture surgery, inpatient or death in patients of lower limbs injuries combined with osteoporosis who combined TCM treatment. Results of statistical analyzes are presented as hazard ratios (HR) and 95% confidence intervals (CI). Differences in the risk of fracture surgery, inpatient or death between groups receiving and not combining TCM were estimated using the Kaplan–Meier method and the log-rank test. Statistical significance was determined using a two-tailed test with a *P*-value <.05.

### 2.5. Ethics approval and consent to participate

Our research was performed in accordance with the World Medical Association Code of Ethics (Declaration of Helsinki). The Institutional Review Board of the Tri-Services General Hospital (TSGH) approved our study and waived the need for individual written informed consent (TSGHIRB No. E202316013).

## 3. Results

**Figure. 1.**
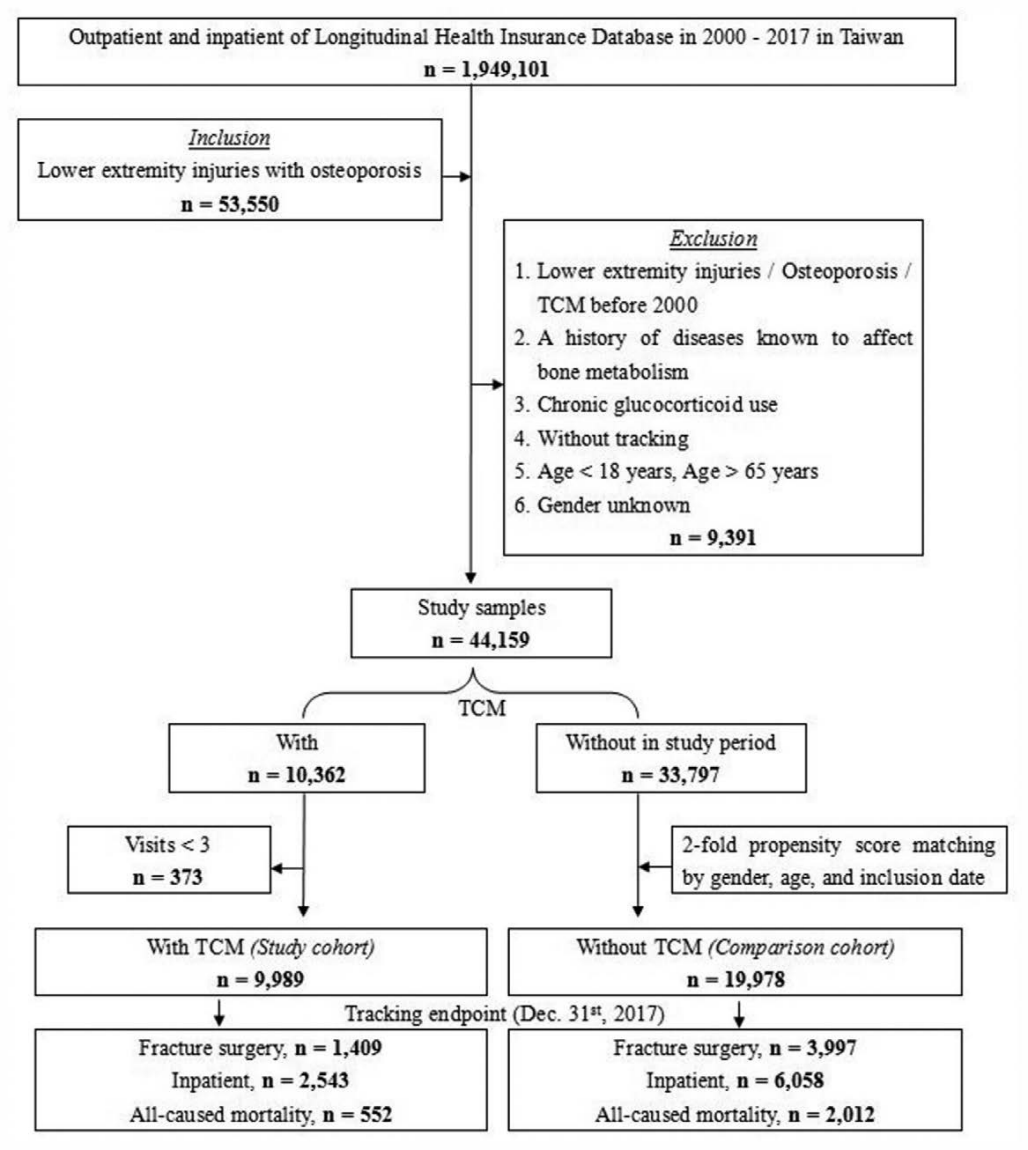
Flowchart of subject selection from the Taiwan National Health Insurance Database. Osteoporosis: ICD-9-CM 733.XX; Lower limbs injuries: ICD-9-CM 820–824, 835, 836, 843, 844, 890, 891, 897, 904, 916, 928, 956, 959; TCM therapy: ≧ 90 days.

**Figure. 2.**
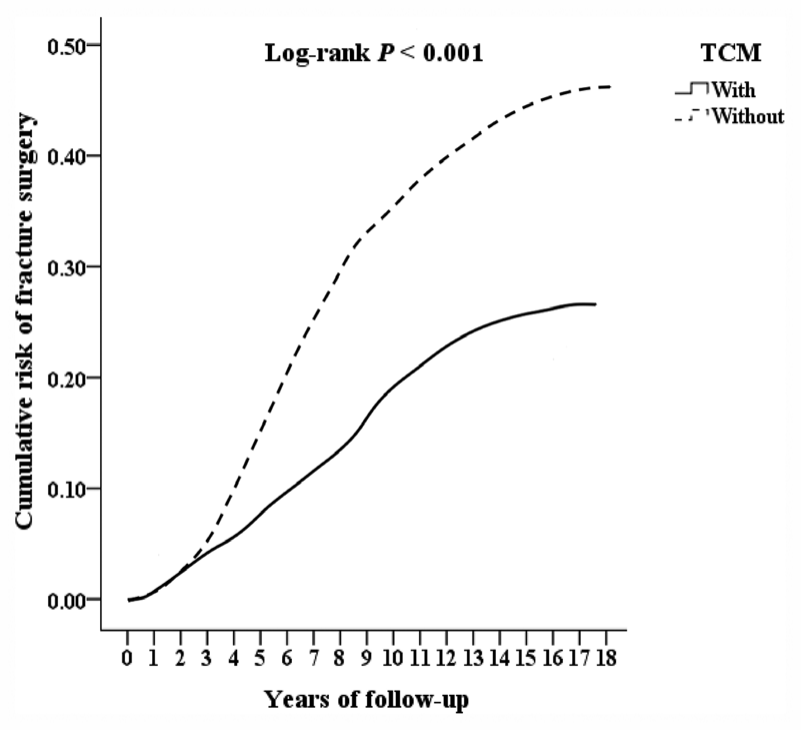
Kaplan–Meier analysis for cumulative risk of fracture surgery in patients of lower limb injuries combined with osteoporosis, as stratified by TCM with log-rank test.

**Figure. 3.**
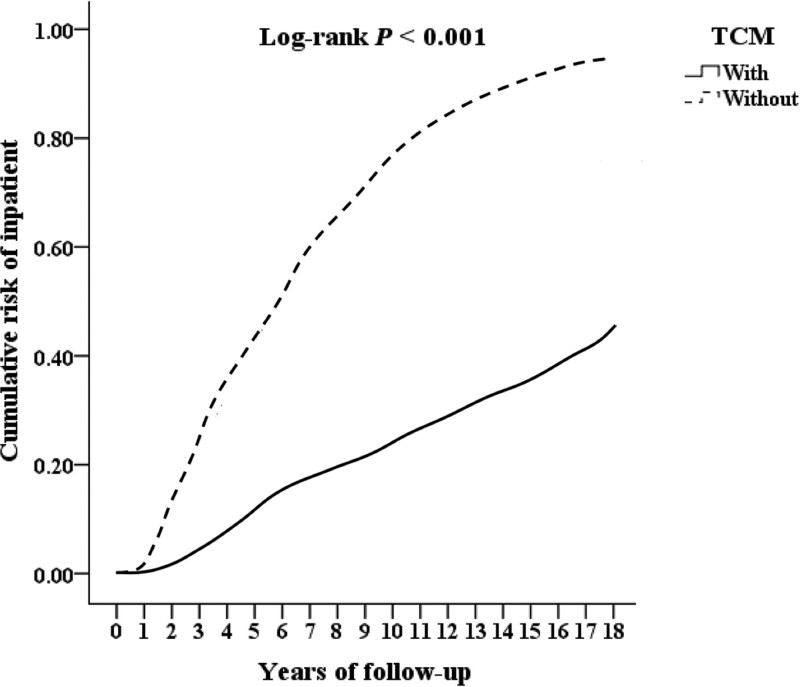
Kaplan–Meier analysis for cumulative risk of inpatient in patients of lower limb injuries combined with osteoporosis, as stratified by TCM with log-rank test.

**Figure. 4.**
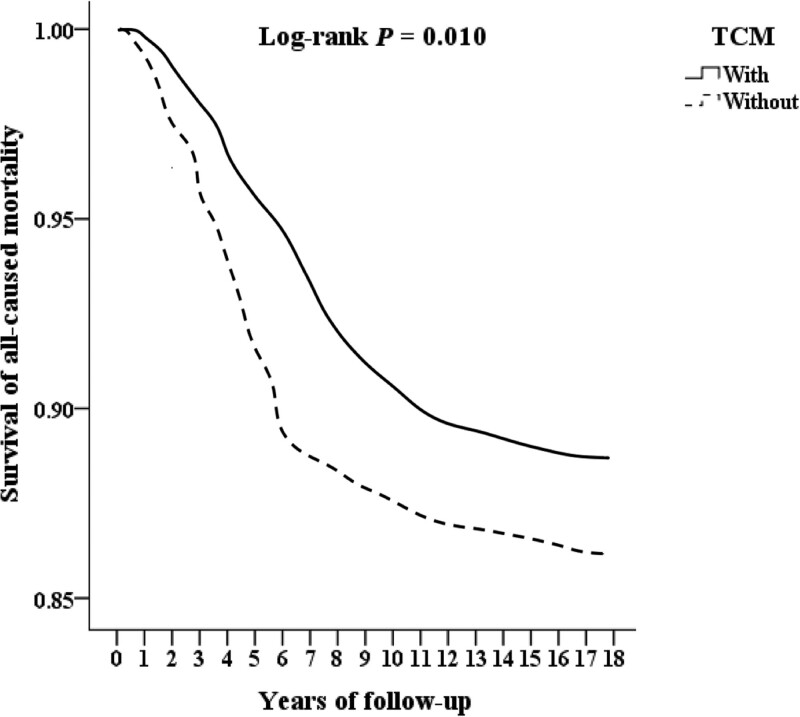
Kaplan–Meier survival analysis of all-cause mortality in patients of lower limb injuries combined with osteoporosis, as stratified by TCM with log-rank test.

We included 53,550 patients of lower limbs injuries combined with osteoporosis, excluding 9391 patients who with lower limbs injuries, osteoporosis or receiving TCM treatment before 2000, patients with known bone metabolic diseases, long-term use of steroids, no follow-up records, younger than 18 years old, and unknown gender, and finally 44,159 patients were included. There were 10,362 patients combined TCM treatment, excluding 373 outpatient follow-up visits <3 times, and finally 9989 patients combined TCM treatment. There were 33,797 people who did not combine TCM treatment, and those who did not combine TCM treatment were matched with gender, age and inclusion date by 2:1 propensity score and finally 19,978 patients without combining TCM treatment in the control group. There were 1049 cases of fracture surgery, 2543 cases of hospitalization, and 552 cases of death. Among those who did not combine TCM treatment, there were 3997 cases of fracture surgery, 6058 cases of hospitalization, and 2012 cases of death, as shown in Figure 1. After 10 years of follow-up, the cumulative incidence of fracture surgery in patients combining TCM treatment seems to be half of that without combining TCM treatment those are shown in Kaplan–Meier analysis with statistically significant (log-rank *P*≤.001, *P*≤.001, *P* = .010), as shown in Figs. 2, 3, and 4.

Table 1 shows that there were 29,967 patients of lower limb injuries combined with osteoporosis enrolled which those including 9435 males (31.48%) and 20,532 females (68.52%), with an average age of 53.09 years old. The average severity of the disease was 0.96, and 1.09 ± 1.14 for those who combined TCM treatment, and 1.04 ± 1.09 for those who did not combine TCM treatment, and there was a statistical difference between the 2 groups (*P* < .001). Table 2 shows that at the end of the study, there were 1409/3997 (14.11/20.01 %) fracture operations, 2543/6058 (25.46/30.32 %) hospitalizations, 552/2012 (5.53/10.07 %) patients died who combined/not combine TCM treatment) (all *P* < .001). The group combining TCM treatment had younger age (*P* = .007) and higher disease severity (*P* < .001), and there was no significant statistical difference in gender. The average years of follow-up and years to outcomes were in Table S1, Supplemental Digital Content, http://links.lww.com/MD/L8. In addition, the factors of fracture, inpatient, all-caused mortality stratified by variables listed in the table by using Cox regression were in Table S2, Supplemental Digital Content, http://links.lww.com/MD/L9.

**Table 1:** Characteristics of study in the baseline.

TCM	Total		With		Without		*P*
Variables	n	%	n	%	n	%	
Total	29,967		9989	33.33	19,978	66.67	
*Gender*							.999
Male	9435	31.48	3145	31.48	6290	31.48	
Female	20,532	68.52	6844	68.52	13,688	68.52	
Age (years)	53.09 ± 20.16		53.06 ± 20.08		53.11 ± 20.20		.840
*Age groups (yrs*)							.999
<40	12,735	42.50	4245	42.50	8490	42.50	
≧40	17,232	57.50	5744	57.50	11,488	57.50	
*Catastrophic illness*							.302
Without	22,538	75.21	7549	75.57	14,989	75.03	
With	7429	24.79	2440	24.43	4989	24.97	
CCI	1.06 ± 1.11		1.09 ± 1.14		1.04 ± 1.09		<.001

*P*: Chi-square/Fisher exact test on category variables and *t*-test on continue variables.

CCI = Charlson comorbidity index.

**Table 2:** Characteristics of study in the endpoint.

TCM	Total		With		Without		*P*
Variables	n	%	n	%	n	%	
Total	29,967		9989	33.33	19,978	66.67	
*Fracture surgery*							<.001
Without	24,561	81.96	8580	85.89	15,981	79.99	
With	5406	18.04	1409	14.11	3997	20.01	
*Inpatient*							<.001
Without	21,366	71.30	7446	74.54	13,920	69.68	
With	8601	28.70	2543	25.46	6058	30.32	
*All-caused mortality*							<.001
Without	27,403	91.44	9437	94.47	17,966	89.93	
With	2564	8.56	552	5.53	2012	10.07	
*Gender*							.999
Male	9435	31.48	3145	31.48	6290	31.48	
Female	20,532	68.52	6844	68.52	13,688	68.52	
Age (yrs)	54.72 ± 19.31		54.29 ± 19.03		54.93 ± 19.45		.007
*Age groups (yrs*)							.044
< 40	12,390	41.35	4211	42.16	8179	40.94	
≧ 40	17,577	58.65	5778	57.84	11,799	59.06	
*Catastrophic Illness*							.572
Without	22,458	74.94	7506	75.14	14,952	74.84	
With	7509	25.06	2483	24.86	5026	25.16	
CCI	1.07 ± 1.12		1.12 ± 1.17		1.05 ± 1.10		<.001

*P:* Chi-square/Fisher exact test on category variables and *t*-test on continue variables.

CCI = Charlson comorbidity index.

Table 3 shows factors affecting Cox regression of fracture surgery, hospitalization, and all-cause mortality. Among fracture surgery patients, we found that patients who combined TCM had a lower proportion of fracture surgery, with an adjusted HR of 0.723 (95 CI = 0.604–0.810, *P* < .001). For those with a higher proportion of fracture surgery, the adjusted HR for males was 1.865 (95 CI = 1.724–1.972, *P* < .001), and the adjusted HR for patients over 40 years old was 1.424 (95 CI = 1.285–1.763, *P* < .001), those with severe diseases had an adjusted HR of 2.463 (95 CI = 1.882–2.860, *P* < .001), and those with a higher Charlson comorbidity index had an adjusted HR of 1.513 (95 CI = 1.489–1.673, *P* < .001). Patients who also combined TCM treatment had a lower risk of hospitalization with an adjusted HR of 0.803 (95 CI = 0.712–0.950, *P* = .001). The patients with a higher proportion of hospitalization were the adjusted HR of males was 1.732 (95 CI = 1.624–1.833, *P* < .001), and the adjusted HR of patients over 40 years old was 1.380 (95 CI = 1.115–1.579, *P* < .001). Those with severe disease had an adjusted HR of 1.986 (95 CI = 1.522–2.430, *P* < .001), and those with a higher Charlson comorbidity index had an adjusted HR of 1.302 (95 CI = 1.267–1.435, *P* < .001). In all-cause mortality, the adjusted HR of patients combining TCM treatment was 0.842 (95 CI = 0.731–0.953, *P* = .007), and the mortality rate was lower. Those with higher all-cause mortality were men whose adjusted HR was 1.964 (95 CI = 1.783–2.165, *P* < .001), and patients over 40 years old whose adjusted HR was 1.670 (95 CI = 1.259–2.075, *P* < .001), those with severe diseases had an adjusted HR of 2.064 (95 CI = 1.562–2.573, *P* < .001), and those with a higher Charlson comorbidity index had an adjusted HR of 1.334 (95 CI = 1.270–1.486, *P* < .001).

**Table 3:** Factors of outcomes by using Cox regression.

Outcomes	Fracture surgery				Inpatient				All-caused mortality			
Variables	aHR	95% CI	95% CI	*P*	aHR	95% CI	95% CI	*P*	aHR	95% CI	95% CI	*P*
*TCM*												
Without	Reference				Reference				Reference			
With	0.723	0.604	0.810	<.001	0.803	0.712	0.950	.001	0.842	0.731	0.953	.007
*Gender*												
Male	1.865	1.724	1.972	<.001	1.732	1.624	1.833	<.001	1.964	1.783	2.165	< .001
Female	Reference				Reference				Reference			
*Age groups (yrs*)												
< 40	Reference				Reference				Reference			
≧ 40	1.424	1.285	1.763	<.001	1.380	1.115	1.597	<.001	1.670	1.259	2.075	<.001
*Catastrophic illness*												
Without	Reference				Reference				Reference			
With	2.463	1.882	2.860	<.001	1.986	1.522	2.430	<.001	2.064	1.562	2.573	<.001
CCI	1.531	1.489	1.673	<.001	1.302	1.267	1.435	<.001	1.334	1.270	1.486	<.001

Location had multicollinearity with urbanization level

aHR = adjusted hazard ratio: adjusted variables listed in the table, CI **=** confidence interval, CCI = Charlson comorbidity index.

Table 4 further shows that the proportion of fracture surgery in patients combining TCM treatment is lower, the adjusted HR is 0.723 (95 CI = 0.604–0.810, *P* < .001), regardless of gender, age and disease severity, the proportion of fracture surgery in patients combining TCM treatment is lower (all *P* < .001). Table 4 goes on to show that the hospitalization rate of patients who combined TCM treatment was lower, the adjusted HR is 0.803 (95 CI = 0.712–0.950, *P* = .001), regardless of gender (Male *P* = .017; Female *P* < .001), age (<40, *P* < .001; ≥40, *P* = .033) and those without or with serious diseases (without *P* < .001; with *P* = .006). Table 4 further shows that the all-cause mortality rate of patients combining TCM treatment is lower, the adjusted HR is 0.842 (95 CI = 0.731–0.953, *P* = .007), regardless of gender (male *P* = .009; female *P* < .001), age (<40, *P* < .001; ≥40, *P* = .024) and those with or without serious diseases (without *P* < .001; with *P* = .038).

**Table 4:** Factors of fracture surgery, inpatient, all-caused mortality stratified by variables listed in the table by using Cox regression.

TCM	Fracture surgery with vs without *(Reference*)				Inpatient with vs without *(Reference*)				All-caused mortality with vs without *(Reference*)			
Stratified	aHR	95% CI	95% CI	*P*	aHR	95% CI	95% CI	*P*	aHR	95% CI	95% CI	*P*
Total	0.723	0.604	0.810	<.001	0.803	0.712	0.950	0.001	0.842	0.731	0.953	.007
*Gender*												
Male	0.753	0.630	0.845	<.001	0.819	0.724	0.962	0.017	0.848	0.735	0.959	.009
Female	0.709	0.592	0.793	<.001	0.793	0.703	0.940	<.001	0.830	0.721	0.943	<.001
*Age groups (yrs*)												
< 40	0.718	0.596	0.803	<.001	0.763	0.674	0.901	<.001	0.813	0.697	0.899	<.001
≧ 40	0.727	0.607	0.814	<.001	0.829	0.736	0.983	0.033	0.860	0.745	0.972	.024
*Catastrophic illness*												
Without	0.716	0.598	0.794	<.001	0.799	0.704	0.942	<.001	0.831	0.720	0.942	<.001
With	0.733	0.608	0.822	<.001	0.813	0.719	0.954	0.006	0.872	0.751	0.989	.038

aHR **=** adjusted hazard ratio: adjusted for the variables listed in Table 3, CI **=** confidence interval, PYs **=** Person-years.

Table 5 shows factors of prognosis among different TCM subgroups by using Cox regression. These factors show the use of TCM in the population, such as the type of TCM treatment such as herbal prescriptions, acupuncture, TCM traumatology, or a combination of herbal prescriptions. The TCM group had lower fracture risk (adjusted HR = 0.723, 95% CI = 0.604–0.810, *P*≤.001), hospitalization risk (adjusted HR = 0.803, 95% CI = 0.712–0.950, *P* = .001) and all-caused mortality risk (adjusted HR = 0.842, 95% CI = 0.731–0.953, *P* = .007). The distribution of herbal formulae with other information were in Table S3, Supplemental Digital Content, http://links.lww.com/MD/L10.

**Table 5:** Factors of prognosis among different TCM subgroups by using Cox regression and Bonferroni correction for multiple comparisons.

Prognosis	TCM subgroup	Population	Events	PYs	Rate	aHR	95% CI	95% CI	*P*
*Fracture surgery*	Without TCM	19,978	3997	209,713.46	1905.93	Reference			
	With TCM	9989	1409	104,902.10	1343.16	0.723	0.604	0.810	<.001
	Herbal formulae only	9510	1335	99,871.72	1336.71	0.720	0.600	0.807	<.001
	Acupuncture only	40	6	420.08	1428.30	0.769	0.642	0.862	<.001
	TCM traumatology only	58	10	609.15	1641.63	0.883	0.738	0.989	.039
	Herbal formulae + Acupuncture	184	28	1932.35	1449.01	0.780	0.651	0.874	<.001
	Herbal formulae + TCM traumatology	197	30	2068.80	1450.12	7.820	0.653	0.876	<.001
	Herbal formulae	9891	1393	103,872.87	1341.06	0.723	0.602	0.810	<.001
	Acupuncture	224	34	2352.43	1445.31	0.779	0.656	0.872	<.001
	TCM traumatology	255	40	2677.95	1493.68	0.805	0.673	0.903	.001
*Inpatient*	Without TCM	19,978	6058	210,873.12	2872.82	Reference			
	With TCM	9989	2543	105,934.76	2400.53	0.803	0.712	0.950	.001
	Herbal formulae only	9510	2418	100,858.26	2397.42	0.802	0.711	0.948	<.001
	Acupuncture only	40	12	424.16	2829.12	0.946	0.838	1.126	.178
	TCM traumatology only	58	18	611.01	2945.94	0.989	0.873	1.157	.136
	Herbal formulae + Acupuncture	184	45	1951.37	2306.07	0.772	0.685	0.918	<.001
	Herbal formulae + TCM traumatology	197	50	2089.96	2392.39	0.800	0.712	0.946	<.001
	Herbal formulae	9891	2513	104,899.59	2395.62	0.799	0.709	0.944	<.001
	Acupuncture	224	57	2375.53	2399.46	0.805	0.714	0.950	.001
	TCM traumatology	255	68	2700.97	2517.61	0.843	0.747	0.995	.045
*All-caused mortality*	Without TCM	19,978	2012	220,558.34	912.23	Reference			
	With TCM	9989	552	110,278.56	500.55	0.842	0.731	0.953	.007
	Herbal formulae only	9510	524	104,990.47	499.09	0.836	0.724	0.952	.002
	Acupuncture only	40	3	441.16	680.03	0.994	0.893	1.195	.125
	TCM traumatology only	58	4	640.92	624.10	0.950	0.811	1.108	.194
	Herbal formulae + Acupuncture	184	10	2031.38	492.28	0.818	0.715	0.931	<.001
	Herbal formulae + TCM traumatology	197	11	2174.63	505.83	0.852	0.740	0.967	.017
	Herbal formulae	9891	545	109,196.48	499.10	0.838	0.728	0.946	<.001
	Acupuncture	224	13	2472.54	525.78	0.882	0.766	1.000	.050
	TCM traumatology	255	15	2815.55	532.76	0.890	0.777	1.012	.062

aHR = adjusted hazard ratio: adjusted for the variables listed in Table 3, CI = confidence interval, PYs = Person-years.

## 4. Discussion

This study is the first using a large database to investigate the effectiveness of combined TCM with an association of lower risk of fracture surgery, inpatient or death in patients of lower limb injuries combined with osteoporosis. According to the results of the study, combined TCM treatment is a therapeutic option that can assist in reducing the harm to human health caused by lower limb injuries combined with osteoporosis. The present study identified factors affecting Cox regression of fracture surgery, inpatient and all-cause mortality with relatively higher risks in male sex, patients older than 40 years, and those with higher disease severity. However, after combining TCM treatment, regardless of gender, age, and disease severity, the risks of fracture surgery, inpatient and all-cause mortality mentioned above will all be significantly reduced.

The skeletal system like many body tissues undergoes metabolism to keep the metabolism of bones in a balanced state.^[14]^ However, such a balance may be deviated due to factors such as physical fitness, gender, aging, nutritional status, acquired diseases, smoking, drinking, less exercise, and even drugs, which will increase the incidence of osteoporosis and even fractures, causing the patient’s quality of life face a severe test.^[15]^ Combined TCM treatment of osteoporosis generally involves 2 aspects at the same time^[16–18]^: one is to analyze the patient’s physique and adjust it and strengthen the bone filling, so that the viscera and meridians can function well, and improve bone density and alleviate the progress of osteoporosis through the effect of TCM in bone density and progression to remission of osteoporosis. Second, to assist in the treatment of the patient’s original disease of osteoporosis, or pain caused by fractures caused by osteoporosis, or posture changes. TCM practitioners will analyze the patient’s condition and choose the appropriate prescription for treatment.

The treatment of TCM is quite common in the Chinese world.^[19]^ It is generally believed that TCM is good at conditioning and mainly treating chronic diseases, while Western medicine is good at treating emergency and severe diseases.^[20]^ In fact, in recent years, the combination of Chinese and Western medicine and emergency treatment have achieved good results.^[21,22]^ Previous studies by our team have shown that the adjuvant treatment of combined TCM can effectively reduce the hospitalization and death risks of diabetic patients with carcinoma in situ in Taiwan.^[12]^ Previous research literature pointed out that TCM treatment of osteoporosis can effectively improve “bone density and improve “pain, joint swelling and other clinical symptoms.”^[23]^ In addition, according to the statistics of the TNHID, it is also found that patients with osteoporosis can “effectively reduce the probability of fractures” after undergoing TCM treatment.^[17]^ The efficacy of TCM in treating osteoporosis has a certain empirical basis, while TCM emphasizes the harmony and operation of all viscera when treating osteoporosis.^[24]^

One study with similar results to ours mentioned that TCM treatment reduces the incidence of fractures in patients with osteoporosis, it explained that after incorporating age, sex and comorbidities into the Cox proportional hazards model, patients who received TCM treatment were more likely than those who did not receive TCM treatment with a lower risk of fracture that adjusted HR is 0.47 (95% CI: 0.37–0.59), and the longer the time of use of TCM, the lower the incidence of fracture.^[17]^ There is a systematic review of randomized controlled trails mentioning that randomized controlled trials were retrieved from 9 different databases, and the meta-analysis included 12 randomized controlled trials, including 1816 patients, comparing TCM with placebo or standard anti-osteoporosis therapy for bone loss that effect of TCM treatment was found to significantly increase bone mineral density of lumbar spine.^[25]^ It can be seen that in the past research on the treatment of osteoporosis with TCM, it was found that TCM has a certain therapeutic effect on patients with osteoporosis.^[25]^

Many studies have also emphasized that weight-bearing exercise and muscle-strengthening exercise can prevent the development of osteoporosis.^[26]^ One of the studies believes that exercise regulates hormones in bones through mechanical loading as a possible underlying mechanism, but the exact mechanism is still unclear.^[27]^ Another study found that bones respond positively to high-intensity progressive resistance training, including muscle strength, balance, and mobility, can reduce the risk of falls.^[28]^ Individual exercise prescriptions must take into account existing bone health to provide specific recommendations for safe and effective exercise, avoiding unnecessary sports injury.^[29]^ So once injured, especially the lower limbs, the doctor will advise the patient to rest more, but the amount of physical activity decreases, and once the time is prolonged, it will cause continuous loss of muscles, which will lead to sarcopenia, which is also aggravated and caused by osteoporosis that one of the important reasons.^[30]^ This article is to explore that once there is a lower limb injury combined with osteoporosis, the severity of the osteoporosis will be aggravated by reducing exercise, and the risk of fracture surgery, inpatient or death can be improved through combined TCM.

This study also has some limitations. First, this is a study using a database which cannot effectively know the relevant blood test, bone mineral density value of the patient and the name of the relevant TCM used. Second, this is a database of patients seeking medical treatment in Taiwan that information is only for the situation in Taiwan. The third, study cannot infer causality, but can only explain possible related reasons. All of these need to be designed in the future related prospective study, which may be able to solve the above limitations.

In conclusions, osteoporosis has become the second most important epidemic worldwide which after cardiovascular disease.^[8]^ How to prevent osteoporosis or treat patients with existing osteoporosis to reduce the risk of harm to health is important, especially patients combine with lower limbs injuries. This study hopes to provide clinicians with the option of combined TCM treatment for patients of lower limbs injuries combined with osteoporosis, so that such patients will be associate with a lower risk of fracture surgery, inpatient or all-cause mortality.

## Acknowledgments

We appreciate the Health and Welfare Data Science Center, Ministry of Health and Welfare (HWDC, MOHW), Taiwan, for providing the National Health Insurance Research Database (NHIRD) and the Teh-Tzer Study Group for Human Medical Research Foundation in Taiwan.

## Author contributions

**Data curation:** Chien-Jung Lin, Li-Jen Tsai, Kuang-Chung Shih.

**Formal analysis:** Chi-Hsiang Chung.

**Writing – original draft:** Yu-Hua Lu, Chieh-Hua Lu.

**Writing – review & editing:** Chieh-Hua Lu, Wu-Chien Chien.

## Supplementary Material






