# Supplementary material for: Effects of combined traditional Chinese medicine therapy in patients of lower limbs injuries with osteoporosis: A retrospective paired cohort study
Source: Medicine (Baltimore). 2023 Dec 8;102(49):e36489. doi: 10.1097/MD.0000000000036489 (PMC10713129; doi:10.1097/MD.0000000000036489)
Supplement: Supplementary file 1 [file medi-102-e36489-s001.docx]

| **Supplement table 1-1. Abbreviation, ICD-9-CM, ICD-10-CM, ATC-Code and definition** | | |
| --- | --- | --- |
|  | **Abbreviation** | **ICD-9-CM / ICD-10-CM / ATC-Code / Definition** |
| **Study population:** |  |  |
| Lower extremity injuries |  | 820 - 824, 835, 836, 843, 844, 890, 891, 897, 904, 916, 928, 956, 959; S70 - S89 |
| Injuries to the hip and thigh |  | 820, 821, 835, 843, 890, 897, 904, 916, 928, 956, 959; S70 - S79 |
| Injuries to the knee and lower leg |  | 822 - 824, 836, 844, 891, 897, 904, 916, 928, 956, 959; S80 - S89 |
| Osteoporosis |  | 733.0; M80, M81 |
| **Intervention:** Traditional Chinese medicine | TCM |  |
| Herbal formulae |  |  |
| Supplemented free wanderer powder |  | Jia-Wei-Xiao-Yao-San |
| Channel-coursing blood-quickening decoction |  | Shu-Jing-Huo-Xue-Tang |
| Spiny jujube decoction |  | Suan-Zao-Ren-Tang |
| Sweet dew beverage |  | Gan-Lu-Yin |
| Stomach-calming powder |  | Ping-Wei-San |
| Pueraria decoction |  | Ge-Gen-Tang |
| Costusrootand amomum six gentlemen decoction |  | Xiang-Sha-Liu-Jun-Zi-Tang |
| Loniceraand forsythia powder |  | Yin-Qiao-San |
| Minor bupleurum decoction |  | Xiao-Chai-Hu-Tang |
| Pinellia heart draining decoction |  | Ban-Xia-Xie-Xin-Tang |
| Acupuncture |  |  |
| TCM traumatology |  |  |
| **Excluding:** |  |  |
| A history of diseases known to affect bone metabolism |  |  |
| Ankylosing spondylitis | AS | 720; M45 |
| Rheumatoid arthritis | RA | 714; M05.9 |
| Paget's disease |  | 731; M88 |
| Hyperthyroidism |  | 242; E05 |
| Hyperparathyroidism |  | 252; E21 |
| Menopause |  | 627; N95 |
| Chronic glucocorticoid | GC | H02AB; use > 60 days in 18 months |
| **Events:** Outcomes |  |  |
| Fracture surgery |  |  |
| Inpatient |  |  |
| All-caused mortality |  | 001 - 999, E800 - E999; A00 - Z99 |
| **Charlson comorbidity index** | CCI |  |

**ATC - Code : Anatomical Therapeutic Chemical – Code**

| **Supplement table 1-2. Years of follow-up** | | | | | |
| --- | --- | --- | --- | --- | --- |
| **TCM** | **Min** | **Median** | **Max** | **Mean ± SD** | ***P*** |
| **With** | 0.02 | 10.29 | 17.85 | 11.26 ± 9.86 |  |
| **Without** | 0.03 | 10.35 | 17.89 | 11.30 ± 9.91 |  |
| **Total** | 0.02 | 10.31 | 17.89 | 11.29 ± 9.89 | 0.741 |
| ***P*: t-test** | | | | | |

| **Supplement table 1-3. Years to outcomes** | | | | | | | | | | | | | | | |
| --- | --- | --- | --- | --- | --- | --- | --- | --- | --- | --- | --- | --- | --- | --- | --- |
| **Prognosis** | **Fracture surgery** | | | | | **Inpatient** | | | | | **All-caused mortality** | | | | |
| **TCM** | Min | Median | Max | Mean ± SD | *P* | Min | Median | Max | Mean ± SD | *P* | Min | Median | Max | Mean ± SD | *P* |
| **With** | 0.35 | 8.30 | 17.42 | 10.07 ± 9.18 |  | 0.39 | 8.45 | 17.52 | 10.23 ± 9.45 |  | 0.90 | 8.90 | 17.64 | 10.41 ± 9.71 |  |
| **Without** | 0.37 | 8.57 | 17.67 | 10.30 ± 9.23 |  | 0.42 | 8.86 | 17.73 | 10.48 ± 9.72 |  | 0.97 | 9.02 | 17.78 | 10.53 ± 9.86 |  |
| **Total** | 0.35 | 8.51 | 17.67 | 10.22 ± 9.21 | 0.042 | 0.42 | 8.82 | 17.73 | 10.40 ± 9.63 | 0.034 | 0.97 | 8.95 | 17.78 | 10.49 ± 9.81 | 0.318 |
| ***P*: t-test** | | | | | | | | | | | | | | | |
