# Supplementary material for: Effects of combined traditional Chinese medicine therapy in patients of lower limbs injuries with osteoporosis: A retrospective paired cohort study
Source: Medicine (Baltimore). 2023 Dec 8;102(49):e36489. doi: 10.1097/MD.0000000000036489 (PMC10713129; doi:10.1097/MD.0000000000036489)
Supplement: Supplementary file 2 [file medi-102-e36489-s002.docx]

| **Supplement table 2. Factors of fracture, inpatient, all-caused mortality stratified by variables listed in the table by using Cox regression** | | | | | | |
| --- | --- | --- | --- | --- | --- | --- |
| **TCM** | **Fracture** | | | | | |
|  | **With** | | | **Without** *(Reference)* | | |
| **Stratified** | **Events** | **PYs** | **Rate (per 10^5^ PYs)** | **Events** | **PYs** | **Rate (per 10^5^ PYs)** |
| **Total** | 1,409 | 104,902.10 | 1,343.16 | 3,997 | 209,713.46 | 1,905.93 |
| **Gender** |  |  |  |  |  |  |
| Male | 465 | 33,029.14 | 1,407.85 | 1,266 | 66,028.14 | 1,917.36 |
| Female | 944 | 71,872.96 | 1,313.43 | 2,731 | 143,685.32 | 1,900.68 |
| **Age groups (yrs)** |  |  |  |  |  |  |
| < 40 | 584 | 44,222.97 | 1,320.58 | 1,620 | 85,859.30 | 1,886.81 |
| ≧ 40 | 825 | 60,679.13 | 1,359.61 | 2,377 | 123,854.16 | 1,919.19 |
| **Catastrophic illness** |  |  |  |  |  |  |
| Without | 1,056 | 78,826.19 | 1,339.66 | 2,989 | 156,954.33 | 1,904.38 |
| With | 353 | 26,075.91 | 1,353.74 | 1,008 | 52,759.13 | 1,910.57 |
| **TCM** | **Inpatient** | | | | | |
|  | **With** | | | **Without** *(Reference)* | | |
| **Stratified** | **Events** | **PYs** | **Rate (per 10^5^ PYs)** | **Events** | **PYs** | **Rate (per 10^5^ PYs)** |
| **Total** | 2,543 | 105,934.76 | 2,400.53 | 6,058 | 210,873.12 | 2,872.82 |
| **Gender** |  |  |  |  |  |  |
| Male | 824 | 33,353.71 | 2,470.49 | 1,925 | 66,392.47 | 2,899.43 |
| Female | 1,719 | 72,581.05 | 2,368.39 | 4,133 | 144,480.65 | 2,860.59 |
| **Age groups (yrs)** |  |  |  |  |  |  |
| < 40 | 1,022 | 44,656.22 | 2,288.59 | 2,476 | 86,332.07 | 2,868.00 |
| ≧ 40 | 1,521 | 61,278.54 | 2,482.11 | 3,582 | 124,541.05 | 2,876.16 |
| **Catastrophic illness** |  |  |  |  |  |  |
| Without | 1,906 | 79,581.06 | 2,395.04 | 4,532 | 157,815.21 | 2,871.71 |
| With | 637 | 26,353.70 | 2,417.12 | 1,526 | 53,057.91 | 2,876.10 |
| **TCM** | **All-cause mortality** | | | | | |
|  | **With** | | | **Without** *(Reference)* | | |
| **Stratified** | **Events** | **PYs** | **Rate (per 10^5^ PYs)** | **Events** | **PYs** | **Rate (per 10^5^ PYs)** |
| **Total** | 552 | 110,278.56 | 500.55 | 2,012 | 220,558.34 | 912.23 |
| **Gender** |  |  |  |  |  |  |
| Male | 175 | 34,720.13 | 504.03 | 634 | 69,442.89 | 912.98 |
| Female | 377 | 75,558.43 | 498.95 | 1,378 | 151,115.45 | 911.89 |
| **Age groups (yrs)** |  |  |  |  |  |  |
| < 40 | 222 | 46,489.25 | 477.53 | 811 | 90,296.66 | 898.15 |
| ≧ 40 | 330 | 63,789.31 | 517.33 | 1,201 | 130,261.68 | 921.99 |
| **Catastrophic illness** |  |  |  |  |  |  |
| Without | 410 | 82,865.26 | 494.78 | 1,505 | 165,069.11 | 911.74 |
| With | 142 | 27,413.30 | 518.00 | 507 | 55,489.23 | 913.69 |
| PYs = Person-years; Adjusted HR = Adjusted Hazard ratio: Adjusted for the variables listed in Table 3.; CI = confidence interval | | | | | | |
