# Supplementary material for: Effects of combined traditional Chinese medicine therapy in patients of lower limbs injuries with osteoporosis: A retrospective paired cohort study
Source: Medicine (Baltimore). 2023 Dec 8;102(49):e36489. doi: 10.1097/MD.0000000000036489 (PMC10713129; doi:10.1097/MD.0000000000036489)
Supplement: Supplementary file 3 [file medi-102-e36489-s003.docx]

| **Supplement table 3. Distribution of herbal formulae** | | | | |
| --- | --- | --- | --- | --- |
| **Herbal formulae** (English name) | **n** | **%** | **Daily dose per prescriptions (g)** | **Duration of prescriptions (days)** |
| Supplemented free wanderer powder | 2,351 | 23.54 | 4.7 ± 1.9 | 10.4 ± 2.9 |
| Channel-coursing blood-quickening decoction | 986 | 9.87 | 4.5 ± 1.8 | 8.2 ± 2.6 |
| Spiny jujube decoction | 974 | 9.75 | 4.6 ± 1.6 | 8.9 ± 2.8 |
| Sweet dew beverage | 835 | 8.36 | 4.8 ± 1.5 | 8.1 ± 2.5 |
| Stomach-calming powder | 765 | 7.66 | 4.2 ± 1.8 | 7.8 ± 2.7 |
| Pueraria decoction | 718 | 7.19 | 4.3 ± 1.7 | 6.9 ± 2.4 |
| Costusrootand amomum six gentlemen decoction | 693 | 6.94 | 4.0 ± 1.5 | 8.9 ± 2.3 |
| Loniceraand forsythia powder | 642 | 6.43 | 4.4 ± 1.6 | 8.5 ± 2.0 |
| Minor bupleurum decoction | 637 | 6.38 | 4.1 ± 1.8 | 7.7 ± 2.4 |
| Pinellia heart draining decoction | 591 | 5.92 | 4.5 ± 1.4 | 7.8 ± 2.6 |
| Others | 797 | 7.98 | 4.3 ± 1.5 | 7.3 ± 1.9 |
| Total | 9,989 | 100.00 | 4.5 ± 1.7 | 8.6 ± 2.5 |
